# Phase III Trials in India: A Comprehensive Analysis of the Clinical Trials Registry-India

**DOI:** 10.7759/cureus.110645

**Published:** 2026-06-11

**Authors:** Ajaya Sahoo, Bikash R Meher, Monalisa Jena, Biswa M Padhy

**Affiliations:** 1 Pharmacology, All India Institute of Medical Sciences, Bhubaneswar, Bhubaneswar, IND

**Keywords:** clinical study, ctri, phase iii, phase iii clinical trial, randomized controlled trial

## Abstract

Introduction: Phase III clinical trials are essential for establishing the safety and efficacy of therapeutic interventions prior to regulatory approval. India has emerged as an important clinical research hub; however, long-term trends in phase III trials registered in the Clinical Trials Registry-India (CTRI) remain inadequately explored.

Methods: A comprehensive analysis of phase III clinical trials registered in CTRI between 2007 and June 2025 was conducted. Trials were categorized into two periods, namely, 2007-2014 and 2015-2025, representing pre- and post-regulatory reform eras. Data regarding randomization, blinding, comparator type, intervention model, sponsorship, study site, intervention agents, geographic scope, and recruitment status were extracted and analyzed using descriptive statistics and chi-squared/Fisher's exact tests.

Results: A total of 1385 phase III clinical trials were analyzed. Randomized controlled trials significantly increased from 360 (88.7%) in 2007-2014 to 928 (94.8%) in 2015-2025 (p<0.001), while non-randomized studies declined. Active-controlled trials increased significantly (234 (57.6%) vs. 608 (62.1%); p=0.025), and parallel-group designs became more common (365 (89.9%) vs. 922 (94.2%); p=0.005). Industry-sponsored trials decreased from 374 (92.1%) to 799 (81.6%), accompanied by increased participation from educational and research institutions (p<0.001). Drug trials remained predominant, although vaccine and biologic/device-based studies increased significantly in the latter period. Recruitment status analysis demonstrated declining completed trials and increasing ongoing or "not yet recruiting" studies, likely reflecting stricter registration practices and pandemic-related disruptions.

Conclusion: Phase III clinical trials registered in CTRI demonstrated substantial improvements in methodological rigor, comparator selection, intervention design, sponsorship diversity, and reporting characteristics during 2015-2025 compared with 2007-2014. These favorable trends are temporally associated with the major Indian and international regulatory reforms, including the New Drugs and Clinical Trials (NDCT) Rules 2019, International Council for Harmonisation's Guideline for Good Clinical Practice (ICH-GCP) updates, and strengthened ethical oversight. Nevertheless, persistent challenges related to trial completion and reporting transparency warrant continued regulatory strengthening.

## Introduction

Phase III clinical trials constitute a critical stage in the drug development process and are designed to evaluate and confirm the efficacy and safety of new interventions in large and heterogeneous patient populations. These trials play an integral role in regulatory decision‑making and the formulation of clinical practice guidelines. Phase III studies are usually comparative in nature, employing either a placebo or an active standard of care as a comparator, and utilize randomized, parallel‑group, and blinded designs to minimize bias and provide robust estimates of the treatment effect [1]. The evidence generated from phase III trials forms the primary basis upon which regulatory agencies such as the Central Drugs Standard Control Organization (CDSCO) grant marketing authorization for new drugs [2]. Although premarketing clinical trials are essential for establishing the benefit‑risk profile of new interventions, inherent limitations such as a relatively small number of study participants, limited study duration, and rigid selection criteria for trial populations make the conduct of well‑designed phase III trials particularly important for generating reliable evidence prior to regulatory approval [3].

India, on account of its large and diverse patient demography, evolving regulatory frameworks, and expanding academic and industry capacities, has emerged as an important hub for clinical research in recent years [4]. There are numerous opportunities for conducting large‑scale clinical trials in the country owing to the high disease burden, the availability of treatment‑naïve patient populations, and the presence of skilled clinical investigators [5]. However, despite accounting for one‑fifth of the world population, less than 5% of global clinical trials are currently conducted in India, indicating that the country's true potential in clinical research remains underutilized [4]. To enhance transparency in the conduct of clinical research and to provide access to trial data to the Indian population in general and researchers in particular, the Clinical Trials Registry-India (CTRI), a free online platform, was launched on July 20, 2007, for the registration of clinical trials conducted in India. Initially initiated as a voluntary measure, the Drugs Controller General of India (DCGI) subsequently made it mandatory for all clinical trials conducted in India to be registered in CTRI since 2009 [6]. It was established with the objective of improving transparency and providing access to trial data to all stakeholders involved in clinical research [7]. The CTRI database contains all important and relevant data of clinical trials such as type of trial, type of intervention, type of study design, public and scientific titles of the study, details about the principal investigator and primary sponsor, countries of recruitment, study sites, name and type of health condition to be studied, phase of clinical trial, target sample size, recruitment status, and other relevant details [8]. CTRI provides a useful dataset to analyze the research landscape of India.

Despite the public availability of the CTRI database for over nearly two decades, there has been limited attempt at systematically evaluating the long‑term secular trends of phase III trials registered on this platform. Previous studies have analyzed specific aspects of clinical trial registration in India, including a review of phase I clinical trials registered between 2008 and 2022 [9], an audit of the impact of mandatory prospective registration on CTRI [10], and a comprehensive analysis of phase IV clinical trials registered in CTRI [11].

To the best of our knowledge, no comprehensive study has been conducted till date to analyze the characteristics of phase III clinical trials registered in CTRI over its entire period of operation. Hence, we carried out a comprehensive review of all phase III clinical trials registered in CTRI from its inception to mid‑2025, as we believed such an analysis would throw light on different dimensions of phase III research conducted in India and may provide a new perspective to all stakeholders. The study focused on design characteristics including randomization and blinding, sponsorship patterns, type of intervention, geographic scope, and recruitment status, with particular attention to trials conducted in oncology, infectious diseases, and lifestyle‑related non‑communicable diseases.

## Materials and methods

Data source

Phase III clinical studies registered with the CTRI site from commencement (2007) to June 15, 2025, were included in the analysis. Phase III studies were identified using the following search terms: "phase 3", "phase 3 clinical trials", "phase III", "phase 3 trials", "phase III trials", and "phase III clinical trials".

Data selection

All studies were downloaded and scrutinized, and eligible studies were then selected for inclusion. All retrieved studies were reviewed by the investigators to confirm eligibility as phase III clinical trials, and duplicate records were excluded. For the final analysis, trials registered between January 1, 2008, and June 15, 2025, were included to ensure consistency of observation across the study period. Studies that did not meet the designation of phase III clinical trials upon review or those with incomplete or ambiguous trial phase information were not included in the final analysis. The dataset was further divided into 2007-2014 (period 1) and 2015-2025 (period 2) to enable comparison of an earlier "lax policy era" versus a later "stringent policy era" in clinical trial governance, aligning the analysis with a mid-2010s inflection point when major regulatory reforms were introduced and robustly implemented in India. In 2014, there were some regulatory changes that occurred, such as introducing stricter compensation rules for trial-related injury/death and mandatory audiovisual recording of informed consent, guidance on placebo-controlled trials, and requirement for providing ancillary care to clinical trial subjects. Regulatory changes were also brought to streamline the process of ethics committee registration and oversight. Regulatory guidelines also clearly suggested a timeline for the reporting of suspected serious adverse events (SAEs) and compensation to be provided due to injury or death related to SAEs.

Data collection

Each registered trial record contained a set of data elements describing the CTRI number, type of trial, health condition, enrollment status of study participants, type of study design, sample size, trial location, lead sponsor, and other relevant information. We extracted all these data describing the common characteristics of the registered phase III trial. The extracted data was recorded in a Microsoft Excel datasheet and later divided into more specific categories.

All relevant data pertaining to study design were retrieved and recorded, including randomization status (randomized or non‑randomized), type of control (placebo‑controlled or active‑controlled), study design (parallel‑group, crossover, or multifactorial), and blinding (blinded or open‑label). Information regarding the trial setting and geographical location was also extracted, including whether the trial was a single‑center or multicenter study and whether recruitment was conducted in India only or at global sites. Data on the type of intervention (drugs, vaccines, or other interventions including devices and indigenous products) were recorded. The type of lead sponsor was categorized as pharmaceutical industry, academic institution (educational institute), research institute, or others (including hospitals, government agencies, and independent investigators). The disease area under investigation was classified into oncology/cancer, lifestyle diseases (such as diabetes mellitus and hypertension), infectious diseases, and others. The recruitment status of each trial was documented as not yet recruiting, open to recruitment, close to recruitment, completed, or terminated.

The investigators independently categorized trials by disease area and intervention type, and any discrepancies were resolved by discussion and consensus.

Statistical analysis

The data collected were entered into Microsoft Excel (Microsoft Corporation, Redmond, Washington, United States) and subsequently analyzed using IBM SPSS Statistics for Windows, Version 30.0 (IBM Corp., Armonk, New York, United States). Descriptive statistics were employed to summarize categorical variables, which were presented as frequencies and percentages. A comparative analysis between the two study periods, that is, 2007-2014 (period 1) and 2015-2025 (period 2), was conducted to evaluate temporal changes in clinical trial characteristics. Associations between categorical variables and study periods were assessed using the Pearson chi-squared test or Fisher's exact test, as appropriate. A two-sided p-value of <0.05 was considered indicative of statistical significance.

Ethical approval

The study was commenced after obtaining approval from the Institutional Ethics Committee of All India Institute of Medical Sciences, Bhubaneswar (approval number: T/IM-NF/Pharm/24/193).

## Results

The detailed characteristics of phase III clinical trials conducted between 2007 and 2025 are presented in Table 1. We evaluated the characteristics of the included studies based on disease categories (cancers, infectious diseases, lifestyle diseases, and others).

**Table 1:** Characteristics of the included studies by disease types

Allocation	2007-2014				2015-2025				Total
	Cancer	Infectious diseases	Lifestyle	Other	Cancer	Infectious diseases	Lifestyle	Other	
Randomized	98	54	77	131	276	102	154	396	1288
Non-randomized	4	4	3	22	0	4	1	20	58
Ambiguous	4	2	3	4	6	5	2	13	39
Blinding									
Blinded	50	32	53	95	100	67	116	310	823
Open	56	28	30	62	182	44	41	119	562
Comparator									
Active-controlled	70	42	44	78	215	61	110	222	842
Placebo-controlled	34	11	35	54	63	38	43	173	451
Missing	2	7	4	25	4	12	4	34	92
Intervention									
Parallel	103	53	79	130	275	100	153	394	1287
Factorial	0	1	0	1	3	1	0	0	6
Crossover	0	0	0	3	1	1	1	3	9
Single arm	2	6	3	19	0	5	1	22	58
NA	1	0	1	4	3	4	2	10	25
Study site									
Single-centered	8	4	0	3	81	2	5	26	129
Multicentered	98	56	83	154	201	109	152	403	1256
Lead sponsors									
Industry	88	57	81	148	153	97	152	397	1173
Educational institute	8	0	0	0	79	3	1	12	103
Research institute	9	3	2	9	38	10	3	19	93
Others	1	0	0	0	12	1	1	1	16
Region									
India	41	38	44	80	134	73	122	215	747
Global	65	22	39	77	148	38	35	214	638
Agents									
Drugs	97	41	78	146	226	58	144	362	1152
Vaccine	2	18	1	2	0	45	2	22	92
Others	7	1	4	9	56	8	11	45	141
Overall status (global)									
Not yet recruiting	2	0	0	0	22	5	8	20	57
Open	20	5	11	27	92	19	13	120	307
Completed	40	21	44	70	13	7	7	30	232
Terminated	11	1	7	9	5	5	6	6	50
Close to recruitment	13	5	1	12	43	4	9	54	141
NA	20	28	20	39	107	71	114	199	598
Overall status (India)									
Not yet recruiting	5	0	2	6	101	19	30	94	257
Open	15	3	5	13	94	16	25	127	298
Completed	46	40	53	89	31	48	68	124	499
Terminated	13	7	11	20	8	10	7	8	84
Close to recruitment	15	4	1	12	47	17	23	75	194
NA	12	6	11	17	1	1	4	1	53

The association between study period and study characteristics is summarized in Table 2.

**Table 2:** Association between study period and study characteristics (n=1385)

	2007-2014 N (%)	2015-2025 N (%)	χ² value	P-value
Randomized	360 (88.7%)	928 (94.8%)	χ²=22.756	<0.001
Non-randomized	33 (8.1%)	25 (2.6%)		
Ambiguous	13 (3.2%)	26 (2.7%)		
Blinding				
Blinded	230 (56.7%)	593 (60.6%)	χ²=1.831	0.176
Open	176 (43.3%)	386 (39.4%)		
Comparator				
Active-controlled	234 (57.6%)	608 (62.1%)	χ²=7.360	0.025
Placebo-controlled	134 (33%)	317 (32.4%)		
Missing	38 (9.4%)	54 (5.5%)		
Intervention				
Parallel	365 (89.9%)	922 (94.2%)	χ²=15.080	0.005
Factorial	2 (0.5%)	4 (0.4%)		
Crossover	3 (0.7%)	6 (0.6%)		
Single arm	30 (7.4%)	28 (2.9%)		
NA	6 (1.5%)	19 (1.9%)		
Study site				
Single-centered	15 (3.7%)	114 (11.6%)	χ²=21.474	<0.001
Multicentered	391 (96.3%)	865 (88.4%)		
Lead sponsors				
Industry	374 (92.1%)	799 (81.6%)	χ²=31.868	<0.001
Educational institute	8 (2%)	95 (9.7%)		
Research institute	23 (5.7%)	70 (7.2%)		
Others	1 (0.2%)	15 (1.5%)		
Region				
India	203 (50%)	544 (55.6%)	χ²=3.580	0.058
Global	203 (50%)	435 (44.4%)		
Agents				
Drugs	362 (89.2%)	790 (80.7%)	χ²=17.451	<0.001
Vaccine	23 (5.7%)	69 (7%)		
Others	21 (5.2%)	120 (12.3%)		
Overall status (global)				
Not yet recruiting	2 (0.5%)	55 (5.6%)	χ²=326.379	<0.001
Open	63 (15.5%)	244 (24.9%)		
Completed	175 (43.1%)	57 (5.8%)		
Terminated	28 (6.9%)	22 (2.2%)		
Close to recruitment	31 (7.6%)	110 (11.2%)		
NA	107 (26.4%)	491 (50.2%)		
Overall status (India)				
Not yet recruiting	13 (3.2%)	244 (24.9%)	χ²=320.135	<0.001
Open	36 (8.9%)	262 (26.8%)		
Completed	228 (56.2%)	271 (27.7%)		
Terminated	51 (12.6%)	33 (3.4%)		
Close to recruitment	32 (7.9%)	162 (16.5%)		
NA	46 (11.3%)	7 (0.7%)		

A total of 1385 clinical trials conducted between 2007 and 2025 were analyzed, revealing significant variation in study characteristics over time (Tables 1-2). Overall, randomized controlled trials predominated 1288 (93%), with a significantly higher proportion observed in the 2015-2025 period compared to 2007-2014 (928 (94.8%) in period 2 vs. 360 (88.7%) in period 1; p<0.001), while non-randomized studies declined (25 (2.6%) vs. 33 (8.1%); p<0.001) during the same period (Table 2). Blinded studies accounted for 823 (59.4%), showing a modest increase over time (593 (60.6%) vs. 230 (56.7%); p=0.176), whereas open-label designs decreased correspondingly (Table 2). Active-controlled trials were most frequent, 842 (60.8%), with a significant rise in the latter period (608 (62.1%) vs. 234 (57.6%); p=0.025), while placebo-controlled trials remained relatively stable (317 (32.4%) vs. 134 (33%)) (Table 2).

In both periods, parallel intervention design was dominant, 1287 (92.9%), increasing significantly over time (922 (94.2%) in period 2 vs. 365 (89.9%) in period 1; p=0.005), whereas single-arm studies declined (28 (2.9%) in period 2 vs. 30 (7.4%) in period 1; p=0.005) (Table 2). During the entire period, most of the studies were multicentered, 1256 (90.7%). However, there was a significant increase in single-centered trials in the latter period (114 (11.6%) in period 2 vs. 15 (3.7%) in period 1; p<0.001) (Table 2).

Industry-sponsored studies accounted for the majority, 1173 (84.7%), of the trials but decreased significantly over time (799 (81.6%) in the latter period vs. 374 (92.1%) in the former period; p<0.001) which was concurrently accompanied by an increase in educational and research institute sponsorship (Table 2). Geographically, studies conducted solely in India accounted for 747 (53.9%), with a non-significant increase in the latter period (544 (55.6%) vs. 203 (50%); p=0.058) (Table 2).

Over the entire period, drug trials predominated, 1152 (83.2%), but their proportion declined significantly over time (790 (80.7%) in period 2 vs. 362 (89.2%) in period 1; p<0.001), while studies involving vaccines (69 (7%) in the latter period vs. 23 (5.7%) in the former period) and other interventions (120 (12.3%) in the latter period vs. 21 (5.2%) in the former period) increased (Table 2).

Analysis of study status revealed substantial shifts; globally, completed studies decreased markedly (57 (5.8%) in period 2 vs. 175 (43.1%) in period 1), while open (244 (24.9%) vs. 63 (15.5%)) and not yet recruiting studies (55 (5.6%) vs. 2 (0.5%)) increased, alongside a notable rise in missing study status (491 (50.2%) in period 2 vs. 107 (26.4%) in period 1; p<0.001) (Table 2). A similar pattern was observed in India, where completed studies declined (271 (27.7%) in period 2 vs. 228 (56.2%) in period 1), while open (262 (26.8%) vs. 36 (8.9%)) and not yet recruiting studies (244 (24.9%) vs. 13 (3.2%)) increased significantly, with a marked reduction in missing status (7 (0.7%) in the latter period vs. 46 (11.3%) in the former period; p<0.001) (Table 2).

## Discussion

Well-designed phase III clinical trials are essential for establishing the safety and efficacy of new drugs before they are marketed. Our analysis of 1385 clinical trials registered over the two periods demonstrated a clear improvement in design and scientific rigor of the trials in the latter period. These changes may be suggestive of positive regulatory reforms in the Indian clinical research environment around the mid-2010s [12,13]. In the latter period, further progressive regulatory amendments, both nationally and internationally, such as the New Drugs and Clinical Trials (NDCT) Rules 2019 and alignment with international regulations such as the International Council for Harmonisation's Guideline for Good Clinical Practice (ICH-GCP) E6 (R2/R3) as well as the Food and Drug Administration (FDA) and European Medicines Agency (EMA) frameworks may also have been responsible for the favorable trend observed [14,15]. Randomization is important for internal validity because it reduces selection bias and confounding [16]. As shown in Table 2, the percentage of randomized controlled trials increased from 360 (88.7%) in period 1 to 928 (94.8%) in period 2, while non-randomized studies decreased from 33 (8.1%) to 25 (2.6%) (p<0.001). Similar upward trends in randomization have been reported in the analyses of ClinicalTrials.gov and European Union registries, particularly under regulatory oversight from the FDA and EMA, where randomized controlled trials are considered essential for regulatory decision-making [17,18]. Blinded trials have increased from 230 (56.7%) to 593 (60.6%), but this change is not statistically significant (p=0.176). These favorable changes may be due to increased awareness and adoption of GCP guideline and robust research methodology among the research community in India, which were largely driven by initiatives of the Indian Council of Medical Research (ICMR) (National Ethical Guidelines Training Programs, Short-Term Courses in Clinical Research Methodology, Good Clinical Practice (GCP) Workshops, Basic Course in Biomedical Research (BCBR), the National Workshops on Research Methodology and Biostatistics) and other organizations.

Active-controlled trials increased significantly from 234 (57.6%) to 608 (62.1%) (p=0.025), whereas missing comparator information reduced significantly from 38 (9.4%) to 54 (5.5%). This trend supports the Declaration of Helsinki (2013 revision) which discourages placebo use when an effective intervention is available [19]. Such trends have also been observed globally, with regulatory agencies increasingly favoring active comparators to ensure patient safety and clinical relevance [20].

Parallel-group design increased from 365 (89.9%) to 922 (94.2%), while single-arm studies dropped from 30 (7.4%) to 28 (2.9%) (p=0.005). This reflects a clear transition toward more rigorous comparative methodologies. The statistical significance and directional consistency of this shift underscore improved adherence to regulatory expectations requiring comparative effectiveness data, particularly in late-phase trials [21].

Interestingly, single-center trials increased from 15 (3.7%) to 114 (11.6%), while multicenter trials remain predominant; this increase probably reflects COVID-19 pandemic-related disruption in research collaboration and activities [22,23].

Industry-sponsored trials reduced from 374 (92.1%) to 799 (81.6%), with educational institutes increasing their share nearly five times (from 8 (2%) to 95 (9.7%)) and research institutes' contribution growing from 23 (5.7%) to 70 (7.2%) (p<0.001). This shift suggests a redistribution of research leadership, fostering academic participation possibly due to a clear regulatory definition of academic clinical trials in the NDCT 2019 [14].

Drug trials continued to be the most common, but their share decreased from 362 (89.2%) to 790 (80.7%) in period 2. Vaccine trials increased from 23 (5.7%) in period 1 to 69 (7%), and "Others" which include devices, biologics, and cell/gene therapies increased significantly from 21 (5.2%) to 120 (12.3%). This shift reflects changing global health priorities, particularly the rapid expansion of vaccine research during the COVID-19 era as well as a surge in biologics trials post-2020, across the world [24,25].

The findings regarding study participants' recruitment status in global trials indicated a substantial decline in completed trials in period 2 (175 (43.1%) to 57 (5.8%)) and a marked increase in the "not yet recruiting" and missing categories (p<0.001). While statistically significant, this pattern likely reflects a combination of factors, including stricter trial registration requirements, real-time registry updates, and pandemic-related disruptions. Importantly, the increase in missing or not available (NA) status (107 (26.4%) to 491 (50.2%)) highlights persistent challenges in reporting completeness, a limitation consistently reported in global registry analyses [26-28].

In contrast, India-specific study recruitment status demonstrated a significant increase in ongoing and recruiting trials in period 2 (not yet recruiting: 13 (3.2%) to 244 (24.9%); open: 36 (8.9%) to 262 (26.8%); p<0.001), accompanied by a decline in completed studies (228 (56.2%) to 271 (27.7%)). This pattern is suggestive of improved trial registration and initiation following regulatory reforms but also indicates potential delays in completion or reporting possibly due to pandemic-related disruption in line with global trials [23,26,27].

Collectively, these findings indicate that the improvements observed in period 2 are associated with major regulatory and guideline changes both nationally and internationally such as ICH-GCP E6(R2), Declaration of Helsinki, ICMR guidelines, NDCT 2019, and stem cell research. While the trajectory of improvement in India mirrors global trends observed in the United States and European Union, disparities in reporting completeness and trial completion highlight the need for continued regulatory strengthening and capacity building. Future efforts should focus on enhancing transparency and integrating advanced risk-based onsite and central monitoring frameworks to sustain and further advance the quality of clinical research in India. The strength of this study is that it has provided a broad perspective on the status of phase III trials in India. Furthermore, it has used the CTRI dataset which is considered the national registry for registration of clinical trials and assessed the data registered for an extended period. However, the study also carries some inherent limitations, that is, it relies entirely on registry information, the accuracy and completeness of which cannot be independently verified. The study also does not assess trial quality, protocol adherence, or reporting completeness, limiting the broader significance of its conclusions.

## Conclusions

Our findings conclude that there was significant improvement in the nature and design of phase III clinical trials registered on CTRI between 2015 and 2025. There were substantial upgrades in allocation concealment, comparator selection, intervention design, sponsor diversity, agent variety, and completeness of reporting compared to the previous period. These favorable changes were influenced by a multitude of national and international regulatory reforms that improved ethics, trial governance, and research activities aligned with global standards. However, there are still certain persisting problems related to trial completion and reporting, along with the failure to disclose outcomes, indicating that more attention is needed to meet the transparency norms set forth by regulatory agencies. To address the above problems, future clinical trials should require mandatory result disclosure within strict timelines on CTRI and global registries, strengthen ethics committee oversight to ensure compliance with reporting obligations, and adopt automated compliance-tracking systems that flag overdue or incomplete submissions.
